# Rationale and Methodology of Reprogramming for Generation of Induced Pluripotent Stem Cells and Induced Neural Progenitor Cells

**DOI:** 10.3390/ijms17040594

**Published:** 2016-04-20

**Authors:** Zuojun Tian, Fuzheng Guo, Sangita Biswas, Wenbin Deng

**Affiliations:** 1 Department of Neurology, the Institute of Guangzhou Respiratory Disease, State Key Laboratory of Respiratory Disease, the First Affiliated Hospital of Guangzhou Medical University, Guangzhou 510120, China; zjtian@ucdavis.edu or tianzuojun9@163.com; 2 Department of Biochemistry and Molecular Medicine, School of Medicine, University of California, Davis, CA 95817, USA; 3 Institute for Pediatric Regenerative Medicine, Shriners Hospitals for Children, Sacramento, CA 95817, USA; fzguo@ucdavis.edu

**Keywords:** reprogramming, dedifferentiation, transdifferentiation, induced pluripotent stem cell, induced neural progenitor cell

## Abstract

Great progress has been made regarding the capabilities to modify somatic cell fate ever since the technology for generation of induced pluripotent stem cells (iPSCs) was discovered in 2006. Later, induced neural progenitor cells (iNPCs) were generated from mouse and human cells, bypassing some of the concerns and risks of using iPSCs in neuroscience applications. To overcome the limitation of viral vector induced reprogramming, bioactive small molecules (SM) have been explored to enhance the efficiency of reprogramming or even replace transcription factors (TFs), making the reprogrammed cells more amenable to clinical application. The chemical induced reprogramming process is a simple process from a technical perspective, but the choice of SM at each step is vital during the procedure. The mechanisms underlying cell transdifferentiation are still poorly understood, although, several experimental data and insights have indicated the rationale of cell reprogramming. The process begins with the forced expression of specific TFs or activation/inhibition of cell signaling pathways by bioactive chemicals in defined culture condition, which initiates the further reactivation of endogenous gene program and an optimal stoichiometric expression of the endogenous pluri- or multi-potency genes, and finally leads to the birth of reprogrammed cells such as iPSCs and iNPCs. In this review, we first outline the rationale and discuss the methodology of iPSCs and iNPCs in a stepwise manner; and then we also discuss the chemical-based reprogramming of iPSCs and iNPCs.

## 1. Introduction

During the past decade, pluripotency has been demonstrated to restore from adult somatic cells through ectopically co-expressing defined transcription factors (TFs) that normally maintain pluripotency in stem cells. This is known as cellular reprogramming [1,2,3]. This technology has put the field back to the limelight and formed the “Holy Grail” of regenerative medicine. On the one hand, this discovery brought forth a paradigm shift in our understanding that the fate of somatic cells is switchable when providing specific reprogramming factors and appropriate environmental stimuli; on the other hand, it paved the way for modeling human diseases by circumventing the critical ethical concerns and immune rejections related to application of embryonic stem cells (ESCs) and potentially substituted ESCs for diverse clinical applications [4]. Since the pioneering study of Takahashi and Yamanaka [1], substantial progress has been made to improve both the efficiency and safety of the iPSC reprogramming. Patient- and disease-specific induced pluripotent stem cells (iPSCs) have been established via viral- or nanocarrier-based method for a number of diseases, such as multiple sclerosis [5], Alzheimer’s disease [6], amyotrophic lateral sclerosis [7], spinal muscular atrophy [8], as well as Down syndrome, Parkinson disease, Huntington’s disease and Duchenne and Becker muscular dystrophy [9]. However, the production of customized iPSCs is still a technical challenge, e.g., the rationale of epigenetic reprogramming and the mechanism of its low induction efficiency must be well documented before it becomes a routine technique.

Moreover, for further clinical applications, iPSCs need to be efficiently differentiated into the desired cell type because pluripotent stem cells, including iPSCs and ESCs, harbor the potential risk of teratoma formation *in vivo* (Table 1) [10,11]. However, the generation of sufficient amounts of differentiated cells from iPSCs for further basic and clinical applications is complicated and time-consuming. To overcome these obstacles, recent studies have prompted investigation into the possibility of reprogramming somatic cells to become target cell type by direct lineage conversion, bypassing the pluripotent state.

A number of publications have reported reprogramming of mouse and human fibroblasts into induced neural progenitor cells (iNPCs) through viral- or chemical-induced method [12,13]. The iNPCs are capable of self-renewing and differentiating into neurons and glial, holding great promise for both biomedical research and potential cell therapy. This lineage-restricted stem cell reprogramming complements the iPSC technology and circumvents the difficulty of differentiating neural cells from iPSCs. It also decreases the risk of immature tumorigenesis after the transplantation of iPSC progeny or their derivative multipotent stem cells due to potential iPSC contamination or incomplete differentiation [10,11].

Since iPSCs were generated in 2006 [1], this technology has been extensively studied from multiple perspectives, making it possible to deduce the rationale of cell fate conversion from iPSC generation although the mechanisms have not been fully understood. In this comprehensive review, we aim to outline the rationale and systematically summarize the methodology of cellular reprogramming in induction of iPSCs and iNPCs from somatic cells, as well as the limitations and pitfalls. In the last section, we also discuss the chemical-based reprogramming of iPSCs and iNPCs. Finally, we briefly discuss future perspectives on cellular transformation for clinical application.

## 2. Rationale of Reprogramming to Induced Pluripotent Stem Cells (iPSCs)

### 2.1. The Nature of Cellular Reprogramming

To date, reprogrammed cells can be generated through the following four approaches: (a) nuclei transfer [25]; (b) cell-to-cell fusion [26]; (c) cell extracts reprogramming [27]; and (d) direct reprogramming [1,2]. Among the above methods, direct reprogramming is highlighted in this review because it provides an avenue to induce a desired cell type just by introducing a set of known TFs to donor cells via epigenetic reprogramming without actually altering the gene sequence [28]. Thus cellular reprogramming is essentially a process to switch a cell fate from a donor cell to a desired cell. The idea to explore the induction of iPSCs initially stemmed from the somatic cell nuclear transfer (SCNT) research [29,30,31], in which the non-split nuclei from quiescent donor somatic cells were transferred into enucleated oocytes in metaphase II. After full reprogramming by the undefined factors in the recipient oocyte cytoplasm, the nuclei restored a totipotent state and finally gave birth to the sheep Dolly [29]. Although Yamanaka’s group initially completed the landmark studies of generating iPSCs from mouse by forced expression of the four TFs: Oct4, Sox2, Klf4, and c-Myc (“OSKM”) [1,2], the human fibroblasts could not be converted into iPSCs by transduction of retroviral OSKM under the culture condition for mouse ESCs [2]. This indicated that without an appropriate extrinsic environment, the human iPSCs would not be produced. Altogether, these data indicated that the induction of reprogrammed cells can only be completed by orchestrated interactions between the intrinsic factors such as expression endogenous genes, and the extrinsic factors including the stimuli from both cytoplasm and extracellular microenvironment.

### 2.2. Intrinsic and Extrinsic Factors during iPSC Reprogramming

IPSCs can only be induced under an optimized conditions defined by a combination of both, intrinsic and extrinsic factors. In the research of generating iPSCs using a doxycycline-inducible lentiviral system with OSKM, the “secondary” iPSCs were generated using only doxycycline treatment from many “secondary” cells from cell line known as NGFP2 line, but not from NNeo line which has very low levels of Sox2 and Klf4. This indicated that under suboptimal levels of intrinsic factors (low levels of Sox2 and Klf4), the “secondary” iPSCs will not be induced by an opportune extrinsic factor (doxycycline treatment); and after additional transduction with Sox2 and Klf4, faithful “secondary” iPSCs were also produced from NNeo lines [32]. Additionally, the cell plating density had a profound effect on iPSC formation, both low and high plating densities could entirely inhibit iPSC colony formation, suggesting that in the case of improper extrinsic factor (cell plating densities), the “secondary” iPSCs will also not be generated by proper intrinsic factors (endogenous pluripotency genes) [32]. Particularly, there was another interesting phenomenon observed in iPSCs induction. The “primary” reprogramming efficiency of human iPSCs from fibroblasts was significantly less than that of mouse cells (~0.002% *vs.* ~0.06%, respectively); however, the reprogramming efficiency between “secondary” human and mouse iPSCs was in the same range (~2% *vs.* ~4%, respectively) [32,33]. These data indicated that the interactions of similar intrinsic factors (endogenous pluripotency genes in “secondary” fibroblasts) and the similar external environment (in cell culture condition) can produce a similar reprogramming efficiency between different species. As a result, the appropriate levels of both intrinsic and extrinsic factors are equally important in the process of cell fate reprogramming, both are indispensable.

### 2.3. Stoichiometric Expression of Endogenous Pluripotency Genes in iPSC Reprogramming

It is now apparent that only when all endogenous pluripotency genes are expressed at optimal levels, then iPSCs can be generated. Cowan *et al.* [34] demonstrated that keratinocyte-derived primary iPSC colonies first appeared at the 18th days, and afterward the reprogramming efficiency appeared to decline with the length of doxycycline exposure. In addition, the “secondary” fibroblasts with high transgenic expression of Sox2 showed only moderate reprogramming efficiency due to increased cell death, and those with very low levels of Sox2 and Klf4 could not be expanded to “secondary” iPSCs [32,33]. Yamaguchi *et al.* (2011) [35] also revealed that Sox2 played a vital role in a dose-dependent manner in producing iPSCs, lower Sox2 expression resulting in higher efficiency in general. Papapetrou *et al.* (2009) [36] further demonstrated that high Oct4 expression and low Sox2 expression led to the highest efficiency; Oct4 activated the mesodermal gene expression and suppressed ectodermal gene expression, and on the contrary, Sox2 facilitated ectodermal gene expression and lowered mesodermal gene transcription [35,36]. Predominantly, the high Oct4 and low Sox2 stoichiometry is probably required throughout the entire process of reprogramming; when all the endogenous genes are expressed, the endogenous Oct4 levels are still high, whereas Sox2 levels remain low [37,38], which was proved to be a “see saw model” of balance to facilitate cells reaching full pluripotent states [39]. Any imbalance of the linage specific factors produced an undesirable fate and failure to become iPSCs.

**Figure 1:**
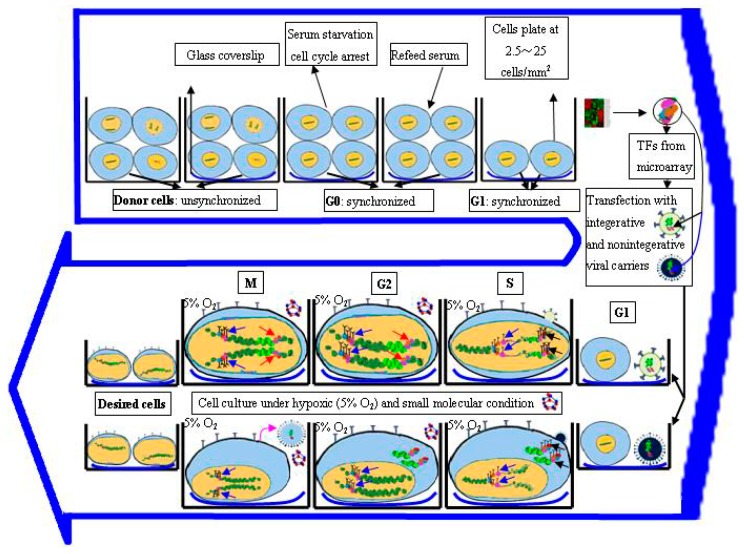
Reprogramming is the process of switching a cell fate from a donor cell to a desired cell, needing orchestrated interactions between the intrinsic factors of endogenous genes and the extrinsic factors from culture microenvironment such as optimal cell plating density, glass coverslip, appropriate small molecules, and hypoxic conditions, e.g., 5% O_2_. The donor cells are induced to cell cycle arrest at Gap 0 (G0) phase by transient serum starvation, and synchronized state to reenter cell cycle after re-feeding with serum. At Gap 1 (G1) phase, the donor cells are transduced with integrative or nonintegrative viral carriers, and returned to a transient Synthesis (S) phase. During S phase the exogenous transcription factors (TFs) from microarray data are transcribed and synthesized (black arrows), initiating endogenous pluripotency/multipotency gene expression (blue arrows). The integrative viral expression is within nucleus, and nonintegrative viral expression is in the cytoplasm. During Gap 2 (G2) phase, nucleosomes mature and histone biogenesis is repressed; the endogenous genes are further expressed to appropriate levels (blue arrows), simultaneously, the extrinsic viral TFs begin to be inhibited (red arrows). During Mitosis (M) phase, many TFs and chromatin binding proteins are ejected from the chromatin; the integrative viruses are gradually silenced, and the nonintegerative viral TFs are gradually removed from host cells (purple arrow). Finally, the desired cells such as induced pluripotent stem cells (iPSCs) and induced neural progenitor cells (iNPCs) are induced [13,26,33,40].

Collectively, the rationale for generating iPSCs can be summarized as such that, it is in nature a process to restore the pluripotency to a somatic cell, which begins with the forced expression of suitable exogenous transgenes/the activity of bioactive chemicals or drugs in opportune extrinsic environment, and more importantly initiates the further reactivation of endogenous pluripotency program and an optimal stoichiometric expression of all the endogenous pluripotency genes, which finally leads to generation of iPSC (Figure 1).

## 3. Methodology of Reprogramming to iPSCs

After the fundamental studies of Yamanaka and coworkers, numerous strategies have been reported to improve the efficiency and safety of iPSC generation. The strategies are mainly explored from the following aspects of iPSC induction: (1) screening of candidate TFs; (2) transduction of donor cells with TFs; (3) activation of endogenous pluripotency genes; (4) regulation of cell cycle status; (5) optimization of cell culture environment; and (6) screening of small molecules, also termed chemical-based reprogramming, which will be discussed in the last section for both iPSCs and iNPCs reprogramming (Figure 1).

### 3.1. Screening of Candidate Transcription Factors (TFs)

Screening for candidate reprogramming factors (TFs) is a key step in direct reprogramming. The logic to select candidate TFs were on the basis of the Yamanaka’s hypothesis that the factors responsible for maintenance of pluripotency in ESCs are also important in inducing pluripotency in somatic cells [1]. The identification of TFs in documented papers generally followed a stepwise process, which fell into two modes: (1) A stochastic mode, in which the candidate TFs were pick from published papers, or by identifying the function of ESC-specific genes. Initially, Yamanaka and coworkers stochastically selected 24 genes as candidate factors from documents to induce pluripotency. Following a comprehensive process of systematic elimination, the combination of bona fide OSKM factors was finally identified sufficient to induce mouse iPSCs [1], and then human iPSCs [2]; (2) In the deterministic mode, TFs were selected from transcriptome database based on DNA microarray data. In 2013, induced mouse oligodendrocyte progenitor cells (iOPCs) were reported by two papers using combinations of reprogramming factors Sox10, Olig2, plus Nkx6.2 or Zfp536 [41,42]. Both of the two studies used microarray data to deterministically identify TFs candidates, which were greatly expressed in OPCs and oligodendrocytes comparable to other neural lineages with knowing roles during oligodendroglia development. Dissecting the functions of the candidate transcription factors is critical in selection process. For iPSC generation, it was known that Oct3/4 and Sox2 act as core transcription factors of pluripotency networks by modulating the expression of pluripotency-associated genes; Myc was known as proto-oncogene which encouraged cellular proliferation and survival; Klf4, similarly to Homeobox Transcription Factor Nanog NANOG, could provoke leukemia inhibitory factor LIF-independent self-renewal [3]. In the process of reprogramming to iPSCs, Oct4 and Sox2 are initially linked with distal elements of silent genes [43] that are mainly targeted by c-Myc and Klf4 at their promoters either by opening chromatin *de novo* or by maintaining the active mark [43,44], which consequently lead to a general expression of genes [43,44,45,46,47]. Single-cells conversion further showed that Oct4 is mainly responsible for cell proliferation, and Sox2 is indicative of cell pluripotency [37].

Some reprogramming genes can be functionally substituted with divergent factors. Soon after the discovery of OSKM, another combination of factors Oct4, Sox2, Nanog, and Lin28 was also found to be able to reprogram somatic cells to pluripotency [48]. Subsequent studies also successively showed that OSKM can be respectively replaced with Nr5a2, Sox1, Esrrb and Glis-1 [49,50,51,52,53,54].

The core reprogramming factors remain highly conserved between different species. The typical example is that OSKM can be used to generate iPSCs form both mouse and human fibroblasts [1,2]; and the single factor Sox2 can directly induce mouse and human iNPCs from respective fibroblasts [21]. These data indicated that the fundamental transcriptional network administrating pluri- and multi-potency is extremely preserved in human and mouse. The comparative studies of gene expression networks further revealed that the conservation of gene expression among human and mouse are mainly in the transcriptional and pathway alteration responses and its constituting substructures still have some divergences [55,56]. Consequently, in the future, direct lineage reprogramming of human, may utilize the same core TFs that of mouse of murine, but the modifying factors and culture conditions may be different.

### 3.2. Transfection of Transcription Factors

**Figure 2:**
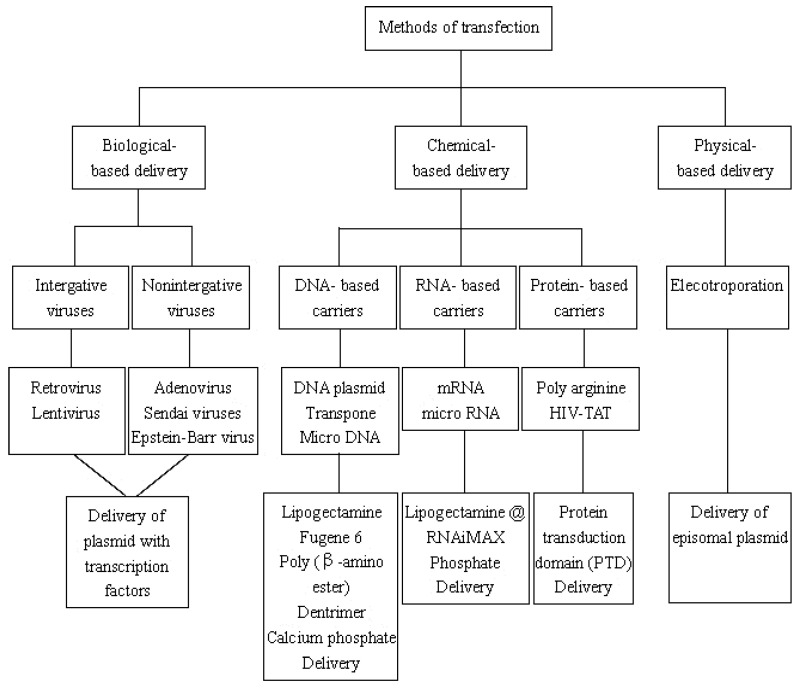
Flow chart outlining the various methods of transfection in induction of iPSCs. These methods are generally classified into biological-, chemical- and physical-based reprogramming.

A variety of methods have been documented to deliver TFs to donor cells for iPSCs generation, most of which relied on biological-, chemical- or physical-based delivery system for transfection (Figure 2). Biological-based delivery uses viral-based carriers including integrative (retrovirus and lentivirus) and nonintegrative viral vectors (adenovirus, Epstein–Barr (EB) and Sendai virus). Chemical-based delivery mainly includes nanocarriers, which contain DNA-, RNA- and protein-based carriers. They are usually regarded as alternatives for viral transduction owing to their delivery of larger transgene, low immunogenicity, easy transfection as well as low risk of gene integration and insertional mutagenesis [57,58]. However, their efficiency and stability remain to be improved. Physical-based delivery involves electroporation, and is seldom reported as successful in reprogramming.

#### 3.2.1. Biological-Based Delivery System

Retrovirus and lentivirus both belong to integrating vectors, however, the latter can infect a broader spectrum of host cells, including actively proliferating cells, terminally differentiated cells and primary cells such as the myocyte or the neuron [28,59,60,61]. Retrovirus was firstly used for iPSCs conversion via introducing OSKM into mouse and human fibroblasts [1,2]. The doxycycline inducible lentiviral gene expression system was used to generate “primary” and “secondary” iPSCs from diverse murine and human somatic cells [32,33,34]. Integrating viruses however, have drawbacks of insertional mutagenesis, reactivation of transgenes and uncontrolled silencing, which prompted nonintegrating viruses to be a safer method for iPSCs generation.

Adenovirus, one kind of nonintegrating carriers, has been regarded as safer method for in induction of mouse and human iPSCs [62,63]. However, it caused rapid multiple organ failure and death of a patient by its high immunogenicity at a clinical trial in 1999 [64]. Sendai virus replicates and transcribes in the cytoplasm of infected cells, and certainly does not integrate into the host genome. Adenovirus has been used to produce iPSCs from human fibroblasts and cord blood cells free of transgene footprints [40,65]. By comparison with other viral carrier, Sendai viral vectors might meet the complex requirements for direct reprogramming, but its enzymes are sometimes not sensitive enough to initiate/complete the reprogramming process [66]. Epstein–Barr virus is far less immunogenic, and has effectively induced human iPSCs free of genomic integration [67], indicating EB viruses may be well-suited for clinical translation. Nevertheless, the clinical applications are obstructed by using viral vectors due to the risk of mutagenesis and immunogenicity, and field has shifted towards the chemical and physical non-integrating strategies.

#### 3.2.2. Chemical-Based Delivery System

DNA-based delivery mainly includes plasmid DNA and minicircle DNA vectors. Plasmid DNA encoding TFs can also be employed for direct conversion of somatic cells. Among them, transposon is a circular plasmid DNA molecule, known as a jumping gene, translocating from one DNA site to another site [68]. Nanoparticles such as Fugene 6, has also been used in generating mouse iPSCs [1]. Minicircle DNA vectors were applied to establish footprint-free iPSC lines [69], but with 10-fold less efficiency than viral-based methods [70].

RNA-based delivery is mostly comprised of synthetic mRNA and micro RNA. Nanoparticles such as Lipofectamine^®^ RNAiMAX, are frequently used to transfect mRNA [28], although at a lower [71] reprogramming rate than that of Sendai, EB and lentiviral reprogramming. However, when using Micro RNA (miRNA) and mRNA transfection, the success rate improved significantly to 73%, similar to other approaches. Additionally, introduction of miR-302s and miR-369s could induce cell reprogramming [72]. Compared to DNA-based delivery, RNA-based carriers can be translated almost instantly in cytoplasm and regarded as a non-gene therapy for not altering the host genome [73]. Nevertheless, they are short half-life, transient expression, and with apparent immunogenicity.

Protein-based delivery, termed as protein transduction domain (PTD), refers to cell penetrating peptides (CPPs), including poly arginine and the human immunodeficiency virus transactivator of transcription (HIV-TAT). Poly arginine consisting of 11 consecutive arginines (abbreviated as 11R) with OSKM were used to induce mouse iPSCs [74]. In 2012, HIV-TAT PTDs were first time utilized to reprogram human iPSCs compared to 11RPTD, indicating TAT-TFs were transcriptionally more active than the corresponding 11R-TFs, but less than retroviral-TFs. Moreover, after linking with cationic liposomes, HIV-TAT transduction was capable of significantly increasing the efficiency by a 1000-fold [75]. These signatures of recombinant TFs may facilitate the clinical application and genetic material-free human iPSCs.

#### 3.2.3. Physical-Based Delivery System

Electroporation: In this technique, an electrical field is employed to increase the permeability of the cell membrane to allow chemicals, drugs, or DNA across the cell membrane via nano-size pores [76]. In 2013, Green and colleagues reported that electroporation method produced faithful iPSCs faster than poly (β-amino ester) [77]. Two years later, another group further revealed that Mesenchymal-to-Epithelial Transition occurring at approximately 6–12 days after electroporation in the process of reprogramming to iPSCs [78]. Electroporation has many advantages such as its technical simplicity, the ability to transiently or stably transfect whole populations of cells and its broad application for transfer of any macromolecule. Unfortunately, there are only few papers successfully using electroporation in cell fate reprogramming to date.

### 3.3. Activation of Endogenous Pluripotency Genes

Activating the expression of endogenous pluripotency genes is a key step in generation of iPSCs. Hotta *et al.* (2008) [79] demonstrated that pluripotent cells, different from somatic cells, have a capability to silence retroviruses by *de novo* DNA methylation and other mechanisms. Partly reprogrammed cells show partial silencing and continued expression of the retroviral OSKM genes, and fully reprogrammed cells show entire reactivation of endogenous pluripotency genes and silencing of all the viral factors. Additionally, the iPSCs induced by diverse nonintegrating deliveries such as Sendai and EB viral vectors or some nanocarriers, also showed reactivation of *OSKM* genes, and the carriers were all gradually eliminated during the process of reprogramming [40,67,71]. In particular, some iPSCs could be induced using only combination of small molecules without exogenous transgenes [17] indicating that the ability of small molecules to activate the endogenous pluripotency genes is different from viruses and nonviral carriers. Overall, faithful reprogramming is not dependent on continuous activation and expression of the exogenous transgenes, but on reestablishment of the autoregulatory loop involving the reactivation and expression of all endogenous pluripotency genes to an opportune level.

### 3.4. Regulation of Cell Cycle Status for Reprogramming

Since the first reports of iPSCs generation [1], a variety of techniques have been used to develop more efficient methods for reprogramming. However, the conversion efficiency is still very low (0.001%–0.1%) [1,2,49,58]. The basis for the low efficiency is weakly understood, but some studies implied the stochastic transfection and genetic heterogeneity of donor cells partially contributed to the results that only a very small fraction of infected cells ultimately converted to pluripotency [1,2,49]. To solve this problem, some studies have exhibited that alleviation of the cell cycle arrest, by promoting S-phase entry, could improve the reprogramming efficiency of iPSCs by achieving synchronization in donor cells [80,81,82] (Figure 1).

The detailed mechanism regarding the influence of cell division on reprogramming remains unclear. However, several papers demonstrated the importance of donor cell cycle in reprogramming [83,84], since it is observed that self-renewal and stemness of iPSCs is reassumed during the process of donor cell division. The iPSCs, similar to ESCs, show an atypical cell cycle structure characterized by a short Gap 1 (G1)-phase and prolonged Synthesis (S) phase, which is intimately linked to the self-renewal and pluripotency and beneficial to the synthesis and expression of pluripotency genes in ESCs compared to differentiated somatic cells [85,86,87]. Little is known about the link between acquisition of stem cell properties and altered cell-cycle structure. A truncated G1-phase might protect ESCs from external induction of differentiation, and prolonged S-phase may be beneficial to synthesize and express the pluripotency of ESCs compared to the differentiate somatic cells [80,81,88]. The active promotion of the transition through S-phase might tolerate epigenetic resetting of the genome and/or encourage proliferation to increase the number of cells accessible for stochastic reprogramming. Facilitation though G1 to S-phase of cell cycle requires the stepwise phosphorylation and inactivation of retinoblastoma [89]. This phosphorylation is adjusted by cyclin dependent kinase (CDK), which needs the binding of cyclins (Cyc) for their functional activation [90]. Belmonte *et al.* (2011) [83] observed a 10-fold increase in the reprogramming efficiency by co-expressing CycD1/CDK4 in BJ fibroblasts, whereas downregulation of CycD2 led to a permanent cell cycle arrest in the donor cells undergoing reprogramming, suggesting that S phase offers an unrivaled opportunity to reset or reprogram gene expression profiles [84]. Chen *et al.* (2012) [82] utilized transient serum starvation (18 h) to induce a reversible cell cycle arrest at Gap 0 (G0) phase. After re-feeding with serum, they harvested a clear enrichment of S-phase in synchronized human dermal fibroblasts (20 h), and finally achieved 1.4% Nanog positive clones, which were 15–20 folds higher than unsynchronized conversion. Taken together, the cell-cycle stage of the donor cells is probably vital for successful reprogramming, and S phase provides a window of opportunity to remodel the pluripotent gene expression profiles.

Regardless of the strategy of cell cycle regulation employed in cellular reprogramming, the underlying machinery at this time is unknown. However, by studying the basic molecular activities of each phase during cell cycle, some cues feasible for the epigenetic remodeling may be found [91]. The cell cycle consists of Gap 1 (G1), Synthesis (S), Gap 2 (G2), and Mitosis (M) phase. Gap 0 (G0) refers to the time when a cell leaves the cycle and quit dividing, which may be a temporary resting period or more permanent. Serum starvation is frequently applied to induce a reversible cell cycle arrest at G0 phase [29,82]. G1, Cells produce RNA and synthesize protein in this time, ensuring everything is ready and activated for DNA synthesis. In S phase, the complete DNA instructions must be duplicated so as to produce two similar daughter cells. This stage is an essential part of cell cycle because many proteins that are involved in epigenetic inheritance such as DNA methyltransferase 1 and chromatin assembly factor-1 CAF-1, and in DNA synthesis such as DNA polymerases, replication protein A and processivity clamp proliferating cell nuclear antigen (RPA, and PCNA respectively), are known to colocalize at “replication foci” during this phase. Additionally, some enzymes, modifying histones, constantly connect with their response elements also in this period [92,93,94,95]. G2 phase serves as checkpoints to make sure everything ready to enter Mitosis (M) phase. If necessary, the cell will make proper amendment of DNA synthesis and other intracellular components. M phase, chromosomes condense, and many transcription factors and chromatin binding proteins are ejected from the chromatin. All of the cell's energy is focused on the complex and orderly division into two similar daughter cells [92]. This implies that transducing at G1 phase leading to transition through S phase may offer opportunity potential window for direct reprogramming to occur.

### 3.5. Optimization of Microenvironment for Reprogramming

Cell fate reprogramming relies not only on the roles of reprogramming genes but also on the influences of external microenvironment (culture conditions). For example variations in oxygen tension, cell-cell contact, paracrine signaling, and extracellular matrix, has received considerable attention in the epigenetic landscape and transcriptome of iPSCs conversion. Yoshida *et al.* (2009) [96] achieved enhanced efficiency in iPSCs reprogramming under the hypoxic condition of 5% O_2_ through retroviral or even non-viral transduction. Their results were confirmed by Liu *et al.* [97] who produced a novel iPSCs under 3% O_2_, which showed efficacy after transplanting them to ischemic stroke model in mouse. Extracellular matrix also seems to play an important role in reprogramming. For example, in reprogramming of iNPCs from mouse and human fibroblasts with Sox2, the morphology of mouse embryonic fibroblasts (MEFs) plated on gelatin coated glass coverslips were drastically changed 2–10 days after sox2-viral transduction, but remained unchanged and failed to reprogram for up to four weeks on gelatin coated plastic dishes [21]. Moreover, the efficiency of generating Nestin^+^/Sox2^+^ colonies from MEFs was greater on poly-l-ornithine/laminin-coated glass coverslips than on gelatin on Day 8 after transduction [21]. Similarly, the cell plating density also has a critical effect on iPSC formation. The plating density at 2.5~25 cells/mm^2^ showed the highest efficiency in induction of “secondary” iPSCs, both lower and higher plating densities could almost inhibit GFP+ colony development, indicating that paracrine factors might be initially required to facilitate iPSC growth, and fundamental cell proliferation will be impeded if cells are contact-inhibited before activation of the transgenes [32]. Altogether, the above-mentioned data suggest that besides reprogramming genes, the culture microenvironment is another determinant in the process of lineage reprogramming.

## 4. Rationale of Reprogramming to iNPCs

The transdifferentiation of iNPCs from fibroblasts is achieved through two different reprogramming techniques. Namely, the “indirect reprogramming” refers to use of the OSKM factors to induce iNPCs from fibroblasts, passing through an intermediate transient state by addition of specific growth factors to the reprogramming medium. “Direct reprogramming” on the other hand, means direct generation of iNPCs from fibroblasts by using lineage-specific transcription factors, bypassing the transient pluripotent stage. Here, we will discuss the rationale of both indirect and direct reprogramming to iNPCs.

“Indirect reprogramming” was first reported by Kim *et al.* (2011) [12]. They initially aimed to establish a more general conversion strategy to produce a broad array of unrelated desired cell types including lineage-committed precursors earlier studies demonstrated that numerous partially or incompletely reprogrammed cells, expressing multiple lineage-specific markers, did not show specific physiological function in the process of iPSC transdifferentiation [1,98], indicating that pluripotency may be only one of many possible outcomes of the four-factor reprogramming, and the eventual result may largely rely on extrinsic signaling conditions. Accordingly, they hypothesized that it may be possible to deliberately switch the early reprogramming process toward a defined cell type by using specific permissive signaling inputs, after which the desired cells could be obtained. Based on this hypothesis, they successfully converted fibroblasts not only into functional iNPCs, but also into spontaneously contracting cardiac cells by temporary expressing the same OSKM factors under different culture conditions. The results suggested that changing the duration of transgene expression and the culture conditions to a transient, plastic developmental state could effectively serve as a cellular platform for reprogramming toward diverse lineages. The study forms a basis of a methodology for interlineage reprogramming into multi- or oligo-potent cells.

Regarding “direct reprogramming” to iNPCs, the rationale is similar to that of transdifferentiation to iPSCs, as depending on the expression of all endogenous multipotent NPC genes, which were reactivated by extrinsic transgenes or chemicals under NPC favorable condition. However, the extrinsic transcription factors appeared to be more flexible than those of iPSCs. The iNPCs could be directly transdifferentiated from somatic cells via either Sox2-based/Sox2 alone, or Oct4-based/Oct4 alone methods, whereas the iPSCs could only be induced by OSKM factors or other functional substitutes.

## 5. Methodology of Reprogramming to iNPCs

**Figure 3:**
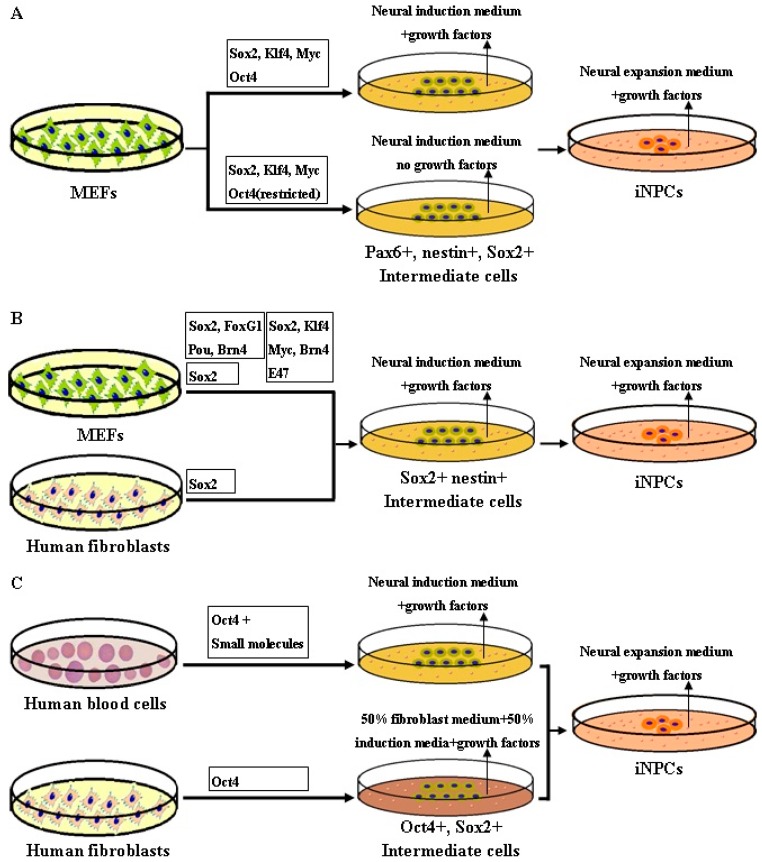
(**A**) A schematic diagram showing iNPC reprogramming from mouse embryonic fibroblasts (MEFs) using four pluripotent factors (OSKM) under neural reprogramming conditions. Neural induction medium with growth factors can deliberately switch the early iPSC reprogramming process toward NPC fate. During the process, the intermediate cells express Pax6, nestin, and Sox2 marks [12]. After restricted Oct4 expression, tripotent iNPCs were obtained without using growth factors in reprogramming medium, indicating the crucial role of the three factors Sox2, Klf4, and c-Myc in iNPCs transdifferentiation [18]; (**B**) the scheme of iNPC conversion from MEF and human fetal fibroblasts using neural specific factors. Sox2 and nestin were expressed in intermediate cells [19,20,21]; (**C**) a schematic showing iNPC conversion from human blood progenitors and fibroblasts via Oct4 factor in neural induction medium with growth factors. The intermediate cells express both Oct4 and Sox2 marks. Tripotent iNPCs can be even induced from adult human fibroblasts using Oct4 alone, indicating iNPC could be mediated through Oct4 trajectory different from Sox2 [22,23].

Several strategies have been explored in the production of iNPCs, among which viral-based or chemical-based approaches have been used successfully (Figure 3). Although the physical-based method involving electroporation has been reported in a few studies to induce integration-free NPCs from human urine cells and porcine fetal fibroblasts [24,99], the nanocarrier-based methods have not yet been extensively explored in iNPC transdifferentiation. A number of studies have demonstrated that the virally-induced reprogramming of iNPCs can be achieved by three principal approaches: expression of four pluripotent factors (OSKM), expression of neural specific factors, or expression of Oct4.

In the indirect conversion to iNPCs by Kim *et al.* (2011) [12], they induced iNPC from doxycycline-induced “secondary” MEFs by reactivation of the typical four iPSC factors (OSKM) with doxycycline under neural reprogramming conditions. However, their iNPC had a few limitations; namely, they quickly lose their capacity to self-renew and to form colonies after 3–5 passages *in vitro*. Moreover they were not tripotent, and could not differentiate into oligodendrocytes. Meyer *et al.* (2005) [100] directly converted adult human fibroblasts into iNPCs by timely restricted expression of all OSKM factors. After 17 days, Sox2-positive neuroepithelial colonies were established, which could only be differentiated into neurons and astrocytes as well. In order to directly achieve faithful iNPCs, Thier *et al.* (2012) [18] used same cocktail of four iPSC factors, while limiting the Oct4 expression after the first five days during the process of transdifferentiation due to the knowledge that NPCs endogenously express only three of the four iPSC transcription factors (c-Myc, Klf4, and Sox2, but not Oct4). Eighteen days after transduction, they harvested neurosphere-like colonies for further characterization, which revealed great similarity between iNPCs and wt NPCs derived from mouse brain, indicating the crucial role of the three factors Sox2, Klf4, and c-Myc in iNPCs transdifferentiation.

In a different study, Lujan *et al.* (2012) [19] obtained tripotent iNPCs from mouse fibroblasts capable of differentiating into neurons, astrocytes and oligodendrocytes using a different four-factor cocktail of lentiviral Sox2, FoxG1, Pou and Brn2. This work evidently confirmed the possibility of achieving self-renewing, multipotent iNPCs using neural-specific reprogramming factors rather than iPSC-related factors. However, this method still carries a reduced risk of tumourigenesis after transplant to animal owing to the constant undifferentiated cells or transgene reactivation; meanwhile it did not show any evidence of deriving neurons and astrocytes from the iNPCs *in vivo*. However, in another study, Han *et al.* (2012) [20] induced a pure population of iNPCs using retroviral Sox2, Klf4, c-Myc, and Brn4 from MEFs, which showed the same epigenetic modifications and multipotentiality of wild typeNPCs. Moreover, the efficiency became higher after infection with a five-factor cocktail47). The iNPCs could be subcultured for more than 130 times, and evidenced capacity of committing both to the neuronal and to the glial lineages, without forming teratomas after transplantation. This study demonstrated once more the critical role of Sox2, Klf4, c-Myc in induction of iNPCs. However, this study focused on mouse iNPCs reprogramming, and the rate of oligodendrocyte differentiation from iNPCs requiring to be enhanced. Thereafter, new method needs to be explored to further improve the multipotency of iNPCs and to induce iNPCs from human somatic cells.

Ring *et al.* (2012) [21] established a homogeneous population of iNPCs from both MEF and human fetal fibroblasts by using only a single factor, retroviral Sox2, which differentiated into neurons, astrocytes, and oligodendrocytes both *in vitro* and *in vivo*. The human iNPCs however, showed morphological and self-renewal properties, which lasted for fewer passages than mouse iNPCs. Fortunately, the human iNPCs were capable of differentiating into three main neural cell types under opportune conditions *in vitro*. Su *et al.* (2013) [101] also direct converted MEF into neural progenitor-like cells by forced growth into 3D spheres on low attachment surfaces and transfected with only a lentival Sox2 factor. These studies highlighted the crucial role of Sox2 as a “master reprogramming gene” for producing iNPCs from differentiated cells.

Mitchell *et al.* (2014) [22] demonstrated that they had directly generated tri-potent neural progenitors from adult human fibroblasts using Oct-4 alone. Human Fib-NPC (Oct-4) could proliferate and express neural progenitor markers, as well as possess the potential to gives rise to three major neural subtypes, astrocytes, oligodendrocytes, and neurons with functional capacity. Their data indicated Oct4 is sufficient for inducing neural conversion from human fibroblasts. Bhatia *et al.* [23] enerated iNPCs from neonatal and adult peripheral blood progenitors using Oct4 + SMAD + GSK-3 inhibitors. Interestingly, expression of Sox2 alone failed to induce hiNPCs under these conditions, suggesting the complexity of the mechanism underlying human iNPC reprogramming.

## 6. Chemical-Based Reprogramming to iPSCs and iNPCs

**Figure 4:**
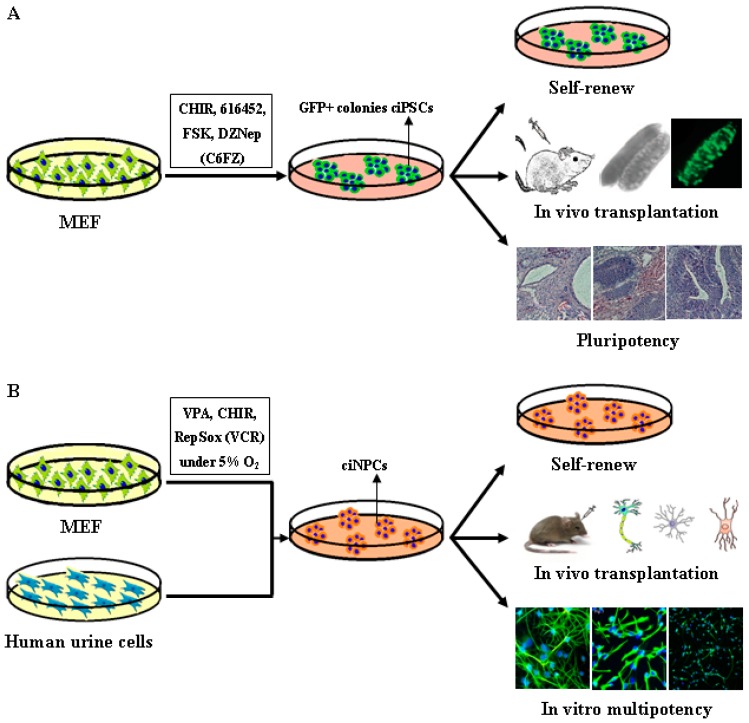
Chemical-based reprogramming to chemically induced pluripotent stem cells (ciPSCs) and chemically induced neural stem cells (ciNSCs): (**A**) Schematics of direct iPSC transdifferentiation from mouse embryonic fibroblasts (MEF) using seven small-molecule cocktail leading to GFP-Oct4 expressing ciPSCs generation. After *in vivo* transplantation, the ciPSCs can differentiate into cells of all three germ layers. After screening, the four small molecules, C6FZ (CHIR99021, 616542, Forskolin and DZNep) were found to be critical in inducing CiPSCs [17]; (**B**) direct ciNSC reprogramming from MEFs and human urinary cells using a three small-molecule cocktail VPA, CHIR99021, and Repsox (VCR) under hypoxic condition (5% O_2_) [13].

Conventional viral-based reprogramming has been proved feasible for generation of iPSCs and iNSCs, however, this method carries the risks of genomic integration, mutagenesis and oncogene expression into host cells [10,11,28]. This has largely prevented this approach from moving towards clinical application. Since this issue also obstructs the therapeutic use of reprogrammed cells such as iPSCs and iNPCs, chemical/small molecule-based reprogramming, as an alternative strategy, has been explored (Figure 4).

In molecular biology and pharmacology, small molecules refer to the organic bioactive compounds with a low molecular weight less than 900 daltons and a size about 10^−9^ m. Most medicines are small molecules. Compared to routine genetic methods, small molecules present several distinct merits: (1) they can rapidly regulate cell functions and even functionally replace some exogenous TFs with higher precision in a temporal and reversible manner; (2) they can be used at adjustable concentrations and combinations with tunable effects; and (3) they are nonimmunogenic, cell permeable, more cost-effective to synthesize, and easier to preserve. Simultaneously, most of chemical-mediated actions are nonspecific; a specific small molecule may work on multiple targets. This feature often presents a challenge for applying them and elucidating the effects in cell reprogramming, however, it is also an opportunity to apply them to some direct lineage reprogramming without knowing the definite reprogramming TFs. These signatures help to improve their efficacy and safety, and be potentially applied in clinical practice.

Most studies demonstrate that the general logic and strategy of using small molecules in transdifferentiation was to replace part or total of the transgenes in different cell fate reprogramming contexts without causing permanent modification to the somatic genome [102,103]. If the epigenetics of target endogenous genes can be regulated by chemicals to enter an active gene status, then there is no need to express ectopic genes [104].

The main function of small molecules in reprogramming is supposedly to activate the silent genes via modulating DNA and histone methylation as well as histone acetylation, so that they are able to undergo reactivation and transcription [105]. According to their functions during cell fate reprogramming, small molecules can be mainly assigned into the following four classes: (a) epigenetic enzyme inhibitors; (b) nuclear receptors agonists; (c) metabolic modulators; and (d) signaling pathway regulators. For more detailed reviews, please refer to Yu *et al.* [103] and Biswas and Jiang [104].

### 6.1. Chemical-Based Reprogramming of Somatic Cells to iPSCs

A vital epigenetic mechanism that controls gene expression is DNA methylation of the promoter. Gene promoter methylation can stably inactivate gene expression by blocking binding of cell-fate-determining TFs to the respective promoter to initiate transcription. Valproic acid (VPA), reported by Huangfu *et al.* [14], was the first small molecule applied to facilitate reprogramming of ciPSC, in which it could replace of the oncogenes c-Myc and Klf4 and enhance the iPSC reprogramming efficiency 100-fold over that of the OSKM method. VPA, trichostatin A (TSA), and butyrate are all histone deacetylases (HDAC) inhibitors; they can improve reprogramming and replace c-Myc via regulating lysine acetylation of histones and loosing chromatin to express transcription [106,107]. Ding *et al.* (2008) [15], revealed that a specific inhibitor for G9a histone methyltransferase (HMT) inhibitor BIX-01294, and BayK (a l-type calcium channel agonist) can enable MEF converting into iPSCs in the presence of Oct4 and Klf4. BIX may functionally facilitate the epigenetic switching of endogenous pluripotency genes from a silenced state to an active transcription state for cell fate reprogramming. Ten-eleven translocation (Tet) enzymes, convert 5-methylcytosine to 5-hydroxymethylcytosine in DNA, as the initial step in activating DNA demethylation [108,109], which not only facilitate Oct4 demethylation and reactivation, but also functionally substitute exogenous Oct4 [110]. Vitamin C was shown to enhance the generation of iPSCs, at least partly owing to its Tet-dependent induction of DNA demethylation [111]. Tranylcypromine, as H3K4 demethylation inhibitors, were shown to promote iPSC generation in the absence of c-Myc [112]. DZNep, as an S-adenosylhomocysteine (SAH) hydrolase inhibitor, can reduce the total levels of histone methylation related with heterochromatin including H3K9, H3K27, and H4K20 methylation [113,114].

Nuclear receptors (NR) are ligand-regulated transcription factors. The major function of NRs is to directly bind DNA and adjust gene expression. As an orphan nuclear receptor, Nr5a2 and its close family member Nr5a1 are capable of both enhancing reprogramming and replacing Oct4 [50]; Esrrb, along with Oct4 and Sox2, can directly activate pluripotency gene Nanog [53]. Together with Nr5a2, RARa/g greatly enhanced reprogramming kinetics and efficiency [50].

Among metabolic modulators, forskolin (FSK), a cAMP agonist, can act as a chemical substitute for Oct4 [17]. Compared with somatic cells, many stem cells depend more heavily on aerobic glycolysis. Many small molecules promote reprogramming via encouraging glycolytic metabolism and acting directly on metabolic pathways, such as fructose 2,6-bisphosphate and Quercetin. On the contrary, a glycolysis inhibitor, 2-deoxy-d-glucose, can inhibit conversion [115].

A majority of small molecules are signaling pathway regulators and thereby modulate cell transdifferentiation. Some signaling pathways even directly link to the pluripotency transcriptional network to positively regulate pluripotent state. Kenpaullone, like CHIR99021, both inhibit GSK-3β and enhance OSKM-based reprogramming, and Kenpaullone can also replace Klf4 [16], which is consistent with the mechanism under WNT stimulation. However since these chemicals show no cell-type specificity, they may be helpful to induce pluripotency by a general open chromatin state, but they won't be capable of directly converting a cell to another differentiated cell type.

Deng and colleagues (2013) were the first group to demonstrated generation of mouse iPSCs by a complete chemical combination of seven small-molecules, with an efficiency of 0.2% (higher than that of Yamanaka’s protocol, 0.01%–0.1%) [17]. After systematic identification, they demonstrated that four chemicals (C6FZ) including CHIR, 616452 (a transforming growth factor-β inhibitor); FSK and DZNep (Z) were indispensable. This study presented the proof of principle that in the context of using small molecules, exogenous “master genes” are dispensable during cell reprogramming, indicating that pure-chemical reprogramming strategy has great potentiality in generating functional reprogrammed cells for clinical cell therapy. If human ciPSC line can be established by complete small molecule method in the near future, it will be tremendously exciting.

### 6.2. Chemical-Based Reprogramming to iNPCs

Wang *et al.* (2013) [24] transfected epithelial-like cells from human urine with episomal vectors carrying Oct4, Sox2, SV40LT, Klf4 and microRNA cluster MIR302–367 through electroporation, and cultured the transfected cells in presence of five chemicals: CHIR99021, PD0325901, A83-01, thiazovivin and DMH1. Fifteen days later, NSC colonies were picked up and differentiated into neurons and glial cells *in vitro*. Ding *et al.* (2014) [116] reported that ectopic expression of Oct4 combined with a chemical combination of A83-01, CHIR99021, NaB, LPA, rolipram, and SP600125, could convert AHDF into hiNPC colonies, that homogeneously expressed the neural stem cell marker PAX6, whereas ectopic expression of Sox2 alone under the same conditions failed to produce hiNPC colonies. Similarly, Bhatia *et al.* [23] demonstrated that iNPCs could be obtained from human blood cells using single-factor Oct4, which could be facilitated by SMAD + GSK-3 inhibitors (SB431542, LDN-193189, Noggin, CHIR99021). Blood-derived iNPCs could differentiate into astrocytes and oligodendrocytes and multiple neuronal subtypes, including dopaminergic (central nervous system related) and nociceptive neurons (peripheral nervous system) *in vitro*. No detectable iNPC-like clusters appeared upon expression of Sox2 alone, and efficiency of iNPC formation was significantly reduced when employed together with Oct4, indicating Sox2-mediated hiNPC transdifferentiation may follow a different trajectory from Oct4-mediated hiNPC transformation.

Pei and colleagues (2014) for the first time demonstrated the generation of chemically induced ciNPCs from both MEFs and human urinary cells by a cocktail of three chemicals: VPA, CHIR99021 and Repsox, (VCR), under a hypoxic condition (5% O_2_) without introducing any exogenous genes [13]. Moreover, the other inhibitors of the same pathways could also induce ciNPCs with similar efficacies. This chemical reprogramming was accompanied by activating the expression of endogenous Sox2. These mouse ciNPCs resembled brain-derived NPCs in both cell identities and multipotency for all three neural cell types *in vitro* and *in vivo*; nevertheless, the human hUC-derived ciNPCs could only differentiate into neurons and astrocytes, not oligodendrocytes. Han *et al.* (2016) [117] also reported an efficient approach to convert MEF into iNPC using a seven combination of small molecules (valproic acid, Bix01294, RG108, PD0325901, CHIR9901, vitamin C, and A83-01). The iNPCs closely resemble wild type NPC and are able to differentiate into astrocytes, functional neurons, and oligodendrocytes *in vitro* and *in vivo*. Consequently, further development and optimization of complete chemical reprogramming to produce tri-potent human ciNPCs will be promising for treating neurological diseases such as multiple sclerosis, Parkinson’s disease and Alzheimer’s disease.

## 7. Concluding Remarks and Future Perspectives

During the past 10 years since the generation of iPSC, significant improvements have been incorporated to increase the efficiency and safety of this technique, making it more amenable mainly for clinical applications in the fields of regenerative medicine, but also for disease modeling and therapeutic discovery. Moreover, the somatic cell reprogramming technology has also opened many avenues for direct lineage reprogramming, which bypasses the iPSC stage and thereby avoids the potential risk of teratoma development. In this context, the iNPCs have been established using viral and nonviral transduction methods. For autologous transplantation, iNPCs could be a great alternative therapy or an essential support to pharmacological treatment. iNPCs should be ideally obtained by avoiding dangerous oncogenic vectors. Compared to viruses, small molecules having their distinct advantages such as easy handling, non-immunogenicity, rapid and reversible effects, might meet the complex requirements for cell fate transformation. However, this technology is far from maturity up to now. Future directions for fate conversion greatly rely on a deeper understanding of mechanisms governing cell identity, plasticity and epigenetic regulations. Despite many challenges, exploiting chemical-based reprogramming for the purpose of generating human ciPSCs or ciNPCs through epigenetic conversion, will make this field an attractive platform to translate the work from bench to bedside for regenerative medicine.

## Figures and Tables

**Table 1:** Summary of viral and chemical reprogramming of induced pluripotent stem cell (iPSC) and induced neural precursor cell (iNPC).

Items	Method	Donor Cells	Duration	Characteristics of iPSCs/iNPCs
*iPSC Studies*				
Takahashi, *et al.* [1]	Retrovirus	Mouse embryonic (MEF) and adult fibroblast	16 days	Could differentiate into all three germ layers *in vitro*
Takahashi, *et al.* [2]	Retrovirus	Adult human fibroblasts	30 days	Could differentiate into cell types of the three germ layers *in vitro*
Hockemeyer, *et al.* [14]	Lentivirus + doxycycline	Primary and secondary human fibroblasts	20–25 days	Primary and secondary human iPSCs
Huangfu, *et al.* [15]	Retrovirus +Valproic acid VPA	Human fibroblasts	30 days	Resemble human ESCs in pluripotency and global gene expression profiles
Shi, *et al.* [16]	Retrovirus+BIX-01294, BayK8644	MEF	14–21 days	Phenotypically and functionally similar to the classic mESCs
Lyssiotis, *et al.* [17]	Retrovirus+ kenpaullone	MEF	20 days	Generate germline-competent chimeras
Hou, *et al.* [12]	CHIR, 616452, FSK and DZNep (C6FZ)	MEF and adult fibroblasts	40 days	Differentiate into tissues of all three germ layers
*iNPC Studies*				
Kim, *et al.* [12]	doxycycline	Doxycycline-inducible secondary MEF	7 days	Lose capacity to self-renew after 3–5 passages *in vitro* and can not differentiate into oligodendrocytes
Their, *et al.* [18]	Retrovirus and lentivirus	MEF	18 days	Differentiate into neurons, astrocytes, and oligodendrocytes.
Lujan, *et al.* [19]	doxycycline-inducible lentiviral + tetO promoter	MEF	24 days	Tripotent *in vitro*, but without evidence of deriving neurons and astrocytes *in vivo*
Han, *et al.* [20]	Retrovirus	MEF	4–5 weeks	Exhibit functionality similar to those of wild-type NPCs *in vitro* and *in vivo*
Ring, *et al.* [21]	Retrovirus	MEF and human fetal fibroblasts	41 days	Differentiate into neurons, astrocytes, and oligodendrocytes
Mitchell, *et al.* [22]	Lentivirus	adult human fibroblasts	14 days	Gives rise to all three major subtypes of neural cells with functional capacity
Lee, *et al.* [23]	Lentivirus + SB431542, Noggin, DN-193189, CHIR99021	Human cord blood or adult peripheral blood cells	10–14 days	Produce astrocytes and oligodendrocytes and multiple neuronal subtypes
Wang, *et al.* [24]	episomal vectors + microRNA + CHIR99021, PD0325901, A83-01, thiazovivin and DMH1	human urine cells	15 days	differentiated into neurons and glial cells *in vitro*
Cheng, *et al.* [13]	VPA, CHIR99021 and Repsox	MEFs and human urinary cells	Mouse 10 days; Human, 20 days	Mouse tripotent iNPCs; Human iNPC could differentiate into neurons and astrocytes
